# “This is fine”: the impact of blowouts on subsequent game performance in the National Hockey League (NHL)

**DOI:** 10.3389/fspor.2023.1241014

**Published:** 2024-01-08

**Authors:** Ravi Chachad, Sean Pradhan, Arman P. Medina

**Affiliations:** ^1^ Department of Medicine, University of Queensland, Brisbane, QLD, Australia; ^2^ School of Business, Menlo College, Atherton, CA, United States; ^3^ Department of Kinesiology, San Jose State University, San Jose, CA, United States

**Keywords:** blowouts, subsequent game performance, National Hockey League (NHL), hot hand phenomenon, cold hand phenomenon, momentum

## Abstract

Blowouts in sports involve large margins of victory or loss between teams and have long been perceived as influencing subsequent performances by athletes, coaches, fans, and other stakeholders. Under the backdrop of the *hot hand* ph enomenon, the current study explores the impact of blowouts on subsequent game performance in the National Hockey League (NHL). Specifically, we examine the potential carryover of a “hot (or cold) hand” on the subsequent game following a large win or loss. In our study, we defined blowouts as outlying goal differentials for regular season games (i.e., a difference of approximately 6 goals between teams in a single game based on 3 standard deviations from the mean goal differential during the sampled period). Using this criterion, data from 285 games over the 2005–06 to 2018–19 NHL regular seasons were gathered for analysis. We performed a series of multiple regressions using blowout goal differential as the main predictor, adjusting for location of the subsequent game, number of time zones from the home base city, whether the subsequent game was a back-to-back, and winning percentages of the team and opponent. Our results revealed no significant over or under performance by teams that either won through a blowout or those that lost by a blowout. Our findings are consistent with previous work in other and similar sports contexts. Practical applications and future directions for research are discussed.

## Introduction

Blowouts in sports are one-sided victories where one team or individual outperforms their opponent by a large margin. These games often attract specific forms of media coverage, which either highlight: (1) the triumphs of the winner [e.g., “Blue Jackets score 10, shut out Canadiens: Four players get two goals, Sergei Bobrovsky makes 30 saves” (1)] or (2) the mistakes of the loser [e.g., “St. Louis Blues’ Ryan O’Reilly rips team after blowout loss to Colorado Avalanche” (2)]. Considering this, much of the previous scholarly research on blowouts has focused on factors related to perceptions of the teams involved. That is, within the existing literature, concepts such as the suitability of “running up the score”, emotional impact, and sportspersonship have been examined.

For instance, the *anti-blowout thesis* proposes that it is unsportspersonlike for teams to continue scoring after victory is secured (3). Dixon contends that it would be rude to humiliate a friend in a recreational racquetball match; furthermore, constantly defeating a child in chess (who is learning to play) would be equally as bad. It would be in the child's best interest to periodically make a weak move or let the child win occasionally. This would encourage the child to enjoy the game and progress as a player. Dixon notes that in a competitive game, there is not an issue for a team to press for a lopsided win, but this does not suggest that a team should run up the score.

As a case in point, on January 13, 2009, Covenant School of Dallas (CSD) beat Dallas Academy 100 to 0 in a girls high school basketball game. A rematch was scheduled, but the Dallas Academy headmaster decided to retire the team for the rest of the season (4). The head of the school and board chair of CSD issued an apology, but the head coach did not and disagreed with this acknowledgement. After claiming his team played the game with “honor and integrity”, CSD's head coach was fired (4, 5). In support of Dixon (3), CSD had the opportunity and capability to not run up the score, but the head coach decided not to. The anti-blowout thesis prescribes that a superior team should conceal its capabilities by easing up.

To that end, blowouts are not necessarily always unsporting, given that it could be argued that teams are treating their opponents with respect by not misrepresenting their abilities. Thus, defeat at the hands of a far stronger opponent should not result in humiliation (3). Dixon uses the example of the United States' 1992 Summer Olympic men's basketball “Dream Team”, where the squad, composed of mainly National Basketball Association (NBA) stars, won their eight Olympic games by an average of 43.8 points. The team also went on to beat six teams in the Olympic qualifying rounds by an average of 51.5 points (6). During a blowout, Dixon also notes that it is not ideal to continue playing star players due to risk of injury. In the NBA, it is common to pull the starters out of the game when a win is imminent [i.e., often referred to as *garbage time* (7)]. The process of pulling starters during garbage time relates to sportspersonship. Some research has argued that this depends on the balance between seriousness of play and playfulness. Feezel (8) contends that in a blowout situation, teams should ease up in a strategic sense, but not from an effort standpoint. In addition, the author suggests that people should not be humiliated by running up the score. Consequently, to avoid disgracing the losing team, teams or individuals should not maximize the margin of victory in a lopsided contest (8).

Research that ties into these concepts stems from the *hot hand phenomenon*, which was originally studied in professional basketball. The hot hand indicates that the probability of making a future shot would be greater after making a previous shot (9). Although a hot hand may be a cognitive illusion that may be labeled as an erroneous belief, getting the ball to a player that is “hot” is a common practice across sports. For instance, according to Attali (10), an NBA player's likelihood of taking the team's subsequent shot after he made a shot increases, as well as the distance of the shot. Moreover, there is a decrease in the probability that a coach will take the player out of the game when this occurs. Thus, the findings from this study shed light on how an athlete's performance can influence both a team's and opponent's sentiments and offensive/defensive schemes around the hot hand or “hot play”, in general. Oftentimes, opponents attempt to play stronger defense on the hot player at the expense of a player without the hot hand. As a result, players perceived as not being “hot” may not be guarded as diligently and may actually have better odds of making an open shot due to the ongoing defensive scheme against the “hot” player (9).

Examinations of the hot hand phenomenon have also been extended to professional hockey goalies by Ding, Cribben, Ingolfsson, and Tran (11). In their study, the authors examined 8 seasons of playoffs, 93 goaltenders, and 48,431 shots to determine whether save performance impacted next-shot save probability. They found that recent positive save performance actually had negative implications of another save at the next opportunity, which is inconsistent with the hot hand phenomenon. Ding et al. hypothesized that motivation could be the causal factor in this occurrence. For example, if a goalie is under performing (based on their average), then they would be more motivated to perform at a higher level. Contrarily, if a goalie is achieving higher than their average, their focus and effort may wane (11).

There are still more inconsistencies found in the literature about the hot hand phenomenon. For example, Green and Zwiebel (12) examined batter statistics from Major League Baseball and found substantial support for the hot hand. They contend that their results differ from other hot hand studies because many are based in the context of basketball as opposed to baseball. A possible explanation for this difference may be attributable to defensive adjustments in basketball and baseball. In basketball, defenses may respond to a hot hand by tightening up the defense on that particular player or changing the defensive scheme (10). Teammates will move the ball to the hot player in basketball and similar sports on several consecutive offensive outings. In baseball, a batter comes to the plate once every nine batters, which could potentially also cool off the hot streak (12). Relatedly, the first offensive evaluation of the hot hand in the National Hockey League (NHL) was of the 2011–12 season by Vesper (13) who found that there is a statistically noteworthy adverse hot hand effect. Vesper argues that it may be the eagerness to take an unwarranted shot after scoring a goal.

Momentum shifts may have the appearance of being influenced by a hot hand. Contrary to popular belief, there is not a positive momentum shift when the losing hockey team initiates a fight with the winning team (14). In this study, the authors obtained data from 458 Division I men's college hockey two-game series, finding that the victory nor the margin of victory in the first game had no predictive influence on the second game. Based on this, they suggest that loss avoidance should hold some weight against momentum. Similarly, Steeger, Dulin, and Gonzalez (15) point out that many studies find that the momentum in sports is not well-supported. In their study, they analyzed momentum in the 2018–19 NHL season, where the St. Louis Blues won the Stanley Cup. The team was propelled by an 11-game win streak during the season and by winning eight of their last ten games. Did the Blues benefit from momentum? Steeger et al. highlight that spectators tend to focus on the streaks instead of the season as a whole, which comparatively is a very small sample. This perception can be construed as momentum, but it is better explained as recency bias. Based on Steeger et al.'s analyses, they found that only one out of all 31 NHL teams benefitted from momentum (i.e., the Anaheim Ducks).

Thus, momentum may exist, but it is at a very rare rate, far below what fans and the media may perceive it to be. Similarly, the motivation of our study is to examine “carryover” of a potential “hot” or “cold hand” effect for teams in subsequent game performance from both game outcome and scoring perspectives. Research using data-driven methods to examine performance of how both winning and losing teams are affected in games after a blowout is scant. Thus, our study adds to the literature by empirically measuring the effects of blowouts on subsequent game performance in the NHL.

## Materials and methods

### Data collection

We collected data from the publicly available, sports database, Hockey-Reference (2020; https://www.hockey-reference.com/leagues/). We extracted box-score statistics from all regular season games played between the 2005–06 and 2018–19 seasons. The 2005–06 season was used as a starting point since it was the first season of play following the 2004–05 NHL lockout, which resulted in the cancellation of the entire 2004–05 season (16). Although the 2012–13 NHL season was shortened by another lockout (17), we included data from games played during this season. The 2019–20 and 2020–21 seasons were excluded from our study due to the unique impact of COVID-19 pandemic, which is not in the scope of the current study. Initially, we compiled data from 16,792 games across this period. Table 1 provides summary statistics for this overall sample.

**Table 1:** Descriptive statistics for overall sample of games.

		Goal differential			
Season	*n*	M	SD	Minimum	Maximum
2005–06	1,230	2.07	1.35	1	9
2006–07	1,230	2.07	1.29	1	8
2007–08	1,230	2.06	1.26	1	9
2008–09	1,230	2.02	1.24	1	9
2009–10	1,230	2.02	1.24	1	8
2010–11	1,230	2.06	1.32	1	8
2011–12	1,230	2.02	1.24	1	9
2012–13	720	2.03	1.18	1	7
2013–14	1,230	1.99	1.22	1	8
2014–15	1,230	2.00	1.19	1	7
2015–16	1,230	2.03	1.17	1	7
2016–17	1,230	2.03	1.23	1	10
2017–18	1,271	2.14	1.28	1	9
2018–19	1,271	2.22	1.30	1	8
Overall	16,792	2.05	1.26	1	10

### Defining blowouts

Among the existing body of literature, there is no widely-accepted threshold of what constitutes a blowout in hockey. Thus, we considered blowouts as outlying goal differentials. Traditionally, statistical outliers refer to values that exceed three standard deviations from a mean value (18). In the current study, we defined a blowout as an outlying instance in margin of victory (loss) greater than or equal to three standard deviations from the mean goal differential during the sample period. Across these seasons, teams won/lost games by approximately 2 goals on average (*M* = 2.05 goals, *SD* = 1.26). Consequently, games with at least a six-goal difference (*M* + 3 *SD* = 5.82 goals) were considered a blowout. As a result, data from 289 games were selected for our analyses.

### Data analysis

All analyses were performed in jamovi [Version 2.3 (19)] and RStudio (Version 2022.02.1; RStudio Team, 2022). First, descriptive statistics were computed to provide an overview of the study sample. Next, point-biserial correlations were conducted to examine the relationships between blowout game differential and subsequent game outcome (win, loss) for both winners and losers. For the purposes of any analyses involving game outcome, overtime losses were equated to losses, similar to Pradhan (20). We also performed Pearson correlations to evaluate the association between blowout game differential and subsequent game differential. For these analyses, we omitted an additional three games from our dataset, as these occurred on the last day of the regular season (*n* = 286 games).

**Figure 1:**
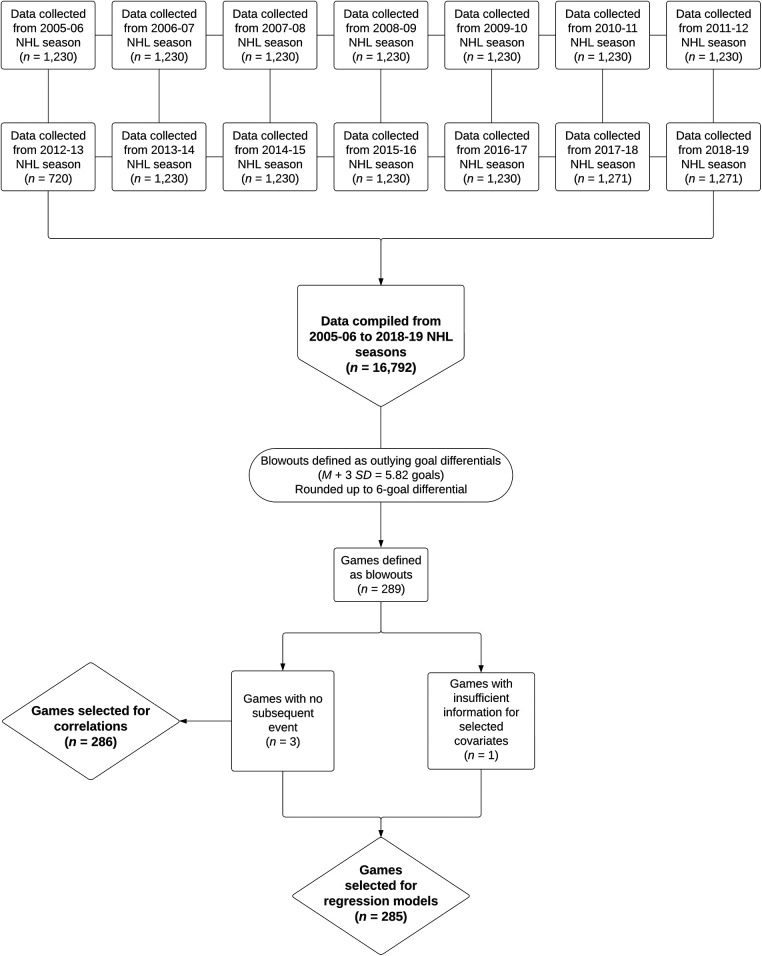
Flowchart of data collection and preparation process.

Ultimately, for our main analyses, a series of multiple regressions were performed using the blowout game goal differential as the main predictor. A binary model was assumed for subsequent game outcome, while a linear model was used for subsequent game goal differential. For each team involved and the ensuing subsequent game opponents, we controlled for the location of the subsequent game (home, away), number of time zones from the home base city, whether the subsequent game was a back-to-back, as well as winning percentages at that point in time. These covariates were selected as they have been shown to impact team performance in a variety of studies (e.g., 21–25). Given that we could not account for all planned covariates for 1 game (i.e., the subsequent game opponent had not played a game and thus had no winning percentage), we omitted this additional game in our regression models (*n* = 285 games). Figure 1 provides a visual representation of the data collection and preparation process.

## Results

### Sample summary statistics

During the sampled period, the average margin of victory (loss) was 6.44 ± 0.77 goals (Range: 6–10 goals) based on our categorization of blowouts. The majority of games played after the blowout were not back-to-back games for both the winning (82.17%) and losing teams (83.51%). On average, approximately 52% of the subsequent games were played on the road for both the winning and losing teams, with nearly three-quarters of subsequent games being played in the same time zone. Following the blowout, winning teams won 53.15% of such games at a margin of 0.24 ± 2.32 goals (Range: −9 to −6 goals), while losing teams went on to win 50.00% of their games with a margin of victory of −0.05 ± 2.31 goals (Range: −6 to −7 goals). Descriptive statistics are summarized in Tables 2, 3.

**Table 2:** Frequencies for characteristics of sampled games.

	*f* (%)	
Variable	Winners	Losers
Subsequent game back-to-back		
Yes	17.83	16.78
* *No	82.17	83.22
Location of next game		
* *Home	48.60	48.25
* *Away	51.40	51.75
Number of time zones away from home		
* *Westward		
* *1 Time zone	6.32	4.84
* *2 Time zone	3.86	3.46
* *3 Time zone	2.11	2.42
* *Same time zone	74.74	73.01
Eastward		
* *1 Time zone	7.37	10.03
* *2 Time zone	2.11	5.19
* *3 Time zone	3.51	1.04
Subsequent game outcome		
* *Win	53.15	50.00
* *Loss	46.85	50.00

**Table 3:** Descriptive statistics for sampled games.

Variable	M	SD	Minimum	Maximum
Goal differential (magnitude of blowout)	6.44	0.77	6	10
Winners				
* *Subsequent game goal differential	0.24	2.32	−9	6
* *Team winning percentage	56.64	13.97	25.00	100.00
* *Subsequent game opponent winning percentage	49.66	15.18	0.00	100.00
Losers				
* *Subsequent game goal differential	−0.05	2.31	−6	7
* *Team winning percentage	45.13	13.83	0.00	83.30
* *Subsequent game opponent winning percentage	49.84	15.85	0.00	100.00

*n* = 286 games.

### Correlations and regression models

The point biserial correlations revealed no meaningful relationship between game differential and subsequent game outcome for winning teams of games that ended in a blowout, *r_pb_*(284) = −0.06, *p* = .32, 95% CI [−0.17, 0.06]. For losing teams, there was also no compelling relationship between blowout game differential and subsequent game outcome, *r_pb_*(284) = 0.01, *p* = .82, 95% CI [−0.10, 0.13]. The Pearson correlations also showed no clear relationship between blowout game differential and subsequent game differential for both winning, *r_pb_*(284) = −0.05, *p* = .44, 95% CI [−0.16, 0.07], and losing teams, *r_pb_*(284) = 0.04, *p* = .54, 95% CI [−0.08, 0.15].

Consistent with the correlations, the binary logistic regressions also revealed no statistically clear association between blowout game differential and subsequent game outcomes for the winning [*b* = −0.10, *SE* = 0.17, *p* = .56, OR* *= 0.91, 95% CI_OR_ (0.65, 1.26)] or losing team [*b* = 0.20, *SE* = 0.17, *p* = .23, OR* *= 1.23, 95% CI_OR_ (0.88, 1.71)]. Furthermore, the multiple linear regression model yielded no statistically meaningful impact of blowout game differential on subsequent game goal differential for the winning [*b* = −0.06, SE = 0.18, *p* = .73, OR* *= 0.94, 95% CI_OR_ (0.66, 1.34)] or losing team [*b* = 0.26, SE = 0.18, *p* = .15, OR* *= 1.30, 95% CI_OR_ (0.91, 1.87)]. Table 4 provides a full summary of the results.

**Table 4:** Regression results for the impact of the magnitude of blowouts on subsequent game outcomes and goal differentials.

	Subsequent game outcome				Subsequent game goal differential			
	Blowout winners (*R^2^* = .08)		Blowout losers (*R^2^* = .08)		Blowout winners (*R^2^* = .12)		Blowout losers (*R^2^* = .07)	
Variable	*χ^2^*	*p*	*χ^2^*	*p*	*χ^2^*	*p*	*χ^2^*	*p*
Goal differential (Magnitude of blowout)	0.34	.56	1.46	.23	0.12	.73	2.04	.15
Covariates								
Location of subsequent game	0.76	.38	2.71	.10	0.51	.47	4.40	.04
Number of time zones away from home for team	3.54	.74	4.77	.57	8.03	.24	2.51	.87
Number of time zones away from home for opponent	5.61	.47	2.01	.92	5.53	.48	1.10	.98
Back-to-back game for team	2.19	.14	0.41	.52	0.67	.41	0.69	.41
Back-to-back game for opponent	2.65	.10	11.36	<.001	4.27	.04	3.98	.05
Team winning percentage	7.76	.01	4.26	.04	8.46	.004	2.76	.10
Opponent winning percentage	4.85	.03	7.64	.01	4.44	.04	6.33	.01

## Discussion

Findings from our study suggest that both winning and losing teams did not under- or over-perform in the game following a blowout. Although the hot hand effect could be pertinent to individual athletes, we did not observe this in our analysis of sampled NHL teams. The implications of this analysis for teams may be that: (1) each game could be independent and (2) blowout results may not determine patterns for the future. As mentioned earlier, Steeger et al. (15) suggest looking at an entire 82-game season to see if there is a pattern of true momentum, which is very rare. Furthermore, our results are consistent with the *hot hand fallacy*, where teams or individuals are not heightened by the possibility of momentum or a streak. As Steeger et al. note, “it is important for owners, coaches, players, and fans to understand the differences between a sequence of random, independent events and a sequence of events that are dependent on each other” (p. 156).

The application of this study and studies like it could be invaluable to both team and individual sports, as well as coaches at all levels. For instance, coaches can use these findings to motivate athletes and teams after a blowout loss, or perhaps even a win. Given the lack of current evidence of a hot hand effect related to blowouts in our study, teams could utilize our results to “bounce back” from losses. As a case in point, teams are often heralded for recovering from blowouts (26), which may speak to the mindset of the players and coaching by the staff to prepare the team for subsequent games. The implications for fans are that they may not have to lament after a loss and can look forward to the subsequent game, as there may not necessarily be a definitive carryover for future games after a blowout. As fan behavior research has shown, there may still be common attitudes felt after a game, such as basking in reflected glory (BIRGing) after a win or cutting off reflected failure following a loss [CORFing (27)]. Nevertheless, teams could encourage fans to avoid behaviors consistent with cutting off future failure [COFFing (28)] following a blowout loss or win by emphasizing that each game may be independent.

One major limitation of the current study relates to our definition of blowouts. Although our decision to utilize the 6-goal threshold was based on classifying blowouts as statistical outliers, future research could consider other cut-offs. It may be useful to examine how players, coaches, and even fans may define blowouts. For instance, large losses against a division rival could be perceived more severely and categorized as a blowout by certain stakeholders. The differentiation of varying levels of goal differentials may be vital in examining team performance in subsequent games. In addition, our analysis only focused on regular season games. Given the importance of post-season play, this work could be extended to investigate whether teams may be susceptible to the effects of blowouts during the playoffs.

Other limitations of our study include not accounting for starting goaltender, player injuries, trades and other roster changes, or performance of key players. Although the covariates we did select have been shown to impact team performance, the utility of each in the current study were mixed. For instance, an opponent playing a back-to-back game meaningfully affected the subsequent game outcome for losers of a blowout and the subsequent goal differential for both winners and losers of a blowout. This did not appear to clearly predict the performance of either the winning or losing team playing a back-to-back game following the blowout. Consequently, the explanatory power of models presented in the current study was limited. Specifically, predictors included in each model explained less than 15% of the variance according to the *R*^2^ values. This may be due to the limited sample of games included in the current study and other potential underlying factors affecting team performance that were not considered. Thus, future directions for research include accounting for some of the aforementioned factors along with other advanced team metrics, such as penalty minutes, possession percentage, shots taken, shots blocked, line changes, active time on ice changes, and other operationalizations of league standing.

Furthermore, there are four major areas of advanced skater statistics that could also be considered, such as Corsi [i.e., a statistic for plus and minus shots on goal for a given player (29], Fenwick [i.e., similar to Corsi, but does include blocked shots (30)], PDO [i.e., a statistic combining shooting and save percentages to calculate how “lucky” or “unlucky” a team is (31)], and Zone Starts [i.e., the amount of faceoffs in any zone that is not neutral (32)]. Future research could also evaluate candidate variables by performing a stepwise regression to construct a model that more parsimoniously explains team performance by beginning with a larger set of potential predictors. Such analyses should be cautious to avoid issues of overfitting statistical models or introducing multicollinearity.

Alternatively, prospective studies could also analyze similar data by splitting the goal differentials into different bands and comparing performance across these levels. This would also enable researchers to investigate interactions between blowout margins and other variables in order to reveal more subtle effects. Furthermore, future research should consider the impact of playing the same starting goaltender in the game following a blowout win or loss. Given that previous research by Ding et al. (11) has found evidence against the hot hand phenomenon in relation to save percentage, it may be worth extending the examination given that goaltenders often play a pivotal role for hockey teams. For instance, any residual effects of a blowout could be dependent on whether the same goalie starts, or another goaltender is used.

Basketball may serve as a suitable milieu to perform a replication of our work because the NBA's season coincides with the NHL's. In addition, both leagues contain the same number of games in their respective seasons. Another area to expand may be from the sport psychology standpoint. A potential debate for the hot hand phenomenon is “feeling good” and the correlation to playing well. In an excerpt of a tweet (33) from Deion Sanders which he originally stated during his playing career, speaks to this point. “U look good u feel good, u *[sic]* feel good u play good”. In the same vein, a study by Vallerand (34) studied 50 male hockey players from the ages of 13–16. The study revolved around positive verbal feedback and intrinsic motivation. The conclusion was that there was a growth in intrinsic motivation with positive performance feedback. One can argue that scoring a goal begets positive performance feedback, which could potentially ignite the perception of a hot hand during certain situations.

Beyond this context, there may be an application of such work related to the hot hand in gambling. For instance, blowouts in sports may not simply affect the athletes involved but may also impact how the oddsmakers set lines and how gamblers behave. Pivotal research by Brown and Sauer (35) posited that the mere belief of the hot hand phenomenon could impact the point-spread betting market. Various other studies have shown that bettors tend to favor hot teams (36, 37), and this has even been shown to transpire among more recent methods of sports gambling [e.g., in daily fantasy sports when setting lineups; (38)]. That said, further research in this area needs to be explored to better contextualize the specific effects of blowouts.

Based on our findings, we conclude that there is not a compelling relationship between the magnitude of a blowout and the subsequent game performance in the NHL. Our findings could generalize to other levels of play [e.g., junior or college hockey (14)] and other complex invasion sports like basketball, football, gridiron football, and rugby. Additional research is necessary to confirm the generalizability of our findings, and we encourage other research to replicate and extend our work. We hope future investigations delve into the intricacies of team performance, as well as the possible psychological ramifications following blowouts to better understand the impact of such games on teams, individual athletes, coaches, and fans.

## Data Availability

Publicly available datasets were analyzed in this study. This data can be found here: via Hockey-Reference (https://www.hockey-reference.com).
